# Oxidative Stress Underlies the Ischemia/Reperfusion-Induced Internalization and Degradation of AMPA Receptors

**DOI:** 10.3390/ijms22020717

**Published:** 2021-01-13

**Authors:** Lindsay M. Achzet, Clara J. Davison, Moira Shea, Isabella Sturgeon, Darrell A. Jackson

**Affiliations:** 1 Department of Pharmaceutical Sciences and Molecular Medicine, Washington State University-Health Sciences, Spokane, WA 99201, USA; lindsay.achzet@wsu.edu; 2 Department of Biomedical Sciences, University of Montana, Missoula, MT 59802, USA; clara.davison@umontana.edu (C.J.D.); moiraflynnshea@gmail.com (M.S.); isabella.sturgeon@umontana.edu (I.S.)

**Keywords:** ischemic/reperfusion injury, AMPA receptor, GluA1, GluA2, reactive oxygen species, oxygen glucose-deprivation/reperfusion (OGD/R), in vitro

## Abstract

Stroke is the fifth leading cause of death annually in the United States. Ischemic stroke occurs when a blood vessel supplying the brain is occluded. The hippocampus is particularly susceptible to AMPA receptor-mediated delayed neuronal death as a result of ischemic/reperfusion injury. AMPA receptors composed of a GluA2 subunit are impermeable to calcium due to a post-transcriptional modification in the channel pore of the GluA2 subunit. GluA2 undergoes internalization and is subsequently degraded following ischemia/reperfusion. The subsequent increase in the expression of GluA2-lacking, Ca^2+^-permeable AMPARs results in excitotoxicity and eventually delayed neuronal death. Following ischemia/reperfusion, there is increased production of superoxide radicals. This study describes how the internalization and degradation of GluA1 and GluA2 AMPAR subunits following ischemia/reperfusion is mediated through an oxidative stress signaling cascade. U251-MG cells were transiently transfected with fluorescently tagged GluA1 and GluA2, and different Rab proteins to observe AMPAR endocytic trafficking following oxygen glucose-deprivation/reperfusion (OGD/R), an in vitro model for ischemia/reperfusion. Pretreatment with Mn(III)tetrakis(1-methyl-4-pyridyl)porphyrin (MnTMPyP), a superoxide dismutase mimetic, ameliorated the OGD/R-induced, but not agonist-induced, internalization and degradation of GluA1 and GluA2 AMPAR subunits. Specifically, MnTMPyP prevented the increased colocalization of GluA1 and GluA2 with Rab5, an early endosomal marker, and with Rab7, a late endosomal marker, but did not affect the colocalization of GluA1 with Rab11, a marker for recycling endosomes. These data indicate that oxidative stress may play a vital role in AMPAR-mediated cell death following ischemic/reperfusion injury.

## 1. Introduction

Stroke is responsible for the death of nearly 140,000 people in the United States annually. Of the estimated 800,000 strokes that occur in the United States each year, approximately 87% are ischemic [1]. Ischemic stroke occurs when a blood vessel in the brain is blocked, hindering the vessel’s ability to provide oxygen and nutrients to brain tissue. While it is necessary to reintroduce blood flow to the infarcted area, this act also results in further damage by inflammation, oxidative stress, and delayed neuronal death (DND) within vulnerable neuronal populations, including CA1 hippocampal pyramidal neurons [2,3]. During ischemia, the lack of energy available disrupts ATP-dependent processes that maintain ionic gradients, which are critical to cellular survival. Disrupting the ionic balance leads to excessive release of neurotransmitters, including glutamate, which are unable to be effectively cleared from synapses [4]. Excessive stimulation of *N*-methyl-*D*-aspartate receptors (NMDARs) by glutamate is a contributing factor to DND [5,6,7,8], but multiple studies have reported that α-amino-3-hydroxy-5-methyl-4-isoxazolepropionic acid receptors (AMPARs) also contribute to DND [9,10,11,12,13,14].

AMPARs are ionotropic glutamate receptors composed of GluA1–4 subunits and can be either homomeric or heteromeric tetramers [15]. A majority of AMPARs are impermeable to Ca^2+^ due to a post-transcriptional modification in the channel pore of the GluA2 AMPAR subunit [16]. Increases in intracellular calcium under physiological conditions regulates many cellular processes, including synaptic plasticity. The overactivation of postsynaptic receptors, such as NMDA receptors and Ca^2+^-permeable AMPARs, leads to an overload of intracellular calcium. This is a key event responsible for a large amount of neuronal death associated with stroke [17]. This process is tightly regulated under physiological conditions, and its dysregulation in pathophysiological conditions, such as ischemic stroke, is catastrophic.

Long-term potentiation (LTP), or strengthening of the synapse, is a tightly regulated process underlying how we form memories and learn. In the simplest form of LTP, AMPARs are inserted into the postsynaptic membrane. During long-term depression (LTD), or weakening of the synapse, AMPARs are removed from the postsynaptic membrane [18]. The incorporation of GluA2-lacking, Ca^2+^-permeable AMPARs into the postsynaptic membrane is important in the induction of LTP [19]. However, when this GluA2 subunit composition switch from GluA2-containing, Ca^2+^-impermeable AMPARs to GluA2-lacking, Ca^2+^-permeable AMPARs becomes long-lasting, the results are detrimental to the cell and contribute to neuronal death in a number of central nervous system pathologies, including stroke [13]. As a result of ischemia/reperfusion, AMPARs undergo a subunit composition switch from Ca^2+^-impermeable, GluA2-containing AMPARs [20], to Ca^2+^-permeable, GluA2-lacking AMPARs. This allows the AMPAR to conduct calcium which, in combination with excessive NMDAR stimulation, exacerbates cell death [21].

During ischemia/reperfusion there is a triphasic burst of superoxide. Upon hypoxic onset, superoxide is produced by the mitochondria as a byproduct from the dysregulated electron transport chain. Next in the hypoxic phase, the cytosolic enzyme xanthine oxidase produces reactive oxygen species, including hydrogen peroxide and superoxide. Once blood flow is restored during the reperfusion phase, NADPH oxidase, a membrane-bound superoxide generator, produces a large burst of superoxide [22]. Additional sources of reactive oxygen species following ischemic/reperfusion injury include monoamine oxidase, catecholamine metabolism, quinone formation, and oxidation of unsaturated fatty acids [23].

In this study, we show that internalization and degradation of GluA2-containing AMPARs following ischemia/reperfusion is mediated through an oxidative stress signaling cascade. U251-MG, a human astroglioma cell line, was utilized in this study due to its high transfection efficiency and consistent response to oxygen-glucose deprivation/reperfusion (OGD/R), an in vitro model for ischemic stroke. U251-MG cells have been used extensively to study various signaling pathways and protein trafficking [24,25,26,27,28,29,30]. U251-MG cells were transiently transfected with fluorescently tagged GluA1 and GluA2, as well as different Rab proteins to examine endocytic/intracellular trafficking of AMPARs following OGD/R. Rab proteins are GTPases that alternate between an inactive GDP-bound state, and an active GTP-bound state. The active GTP-bound state of Rab proteins allows them to interact with downstream effectors [31]. Previous studies have utilized pHluorin-tagged plasmids to observe AMPAR trafficking [32,33], but these tags have proven problematic [34]. In order to clarify the trafficking and intracellular localization of GluA1 and GluA2 AMPAR subunits following OGD/R, we utilized various Rab proteins as biomarkers. Rab5 is localized to early endosomes [35]. Rab7 is localized to late endosomes destined for lysosomal degradation [36]. Rab11 is localized to recycling endosomes that undergo exocytosis [37]. To examine whether superoxide plays a role in AMPAR trafficking following OGD/R, we pretreated the transfected U251-MG cells with Mn(III)tetrakis(1-methyl-4-pyridyl)porphyrin (MnTMPyP), a cell-permeable superoxide dismutase mimetic, and examined the endocytic trafficking of GluA2 and GluA2 AMPAR subunits following OGD/R. Interestingly, we identified oxidative stress-dependent differential trafficking patterns between GluA1 and GluA2 AMPAR subunits following OGD/R.

## 2. Results

### 2.1. OGD/R Did Not Alter Cell Viability in U251-MG Cells

**Figure 1:**
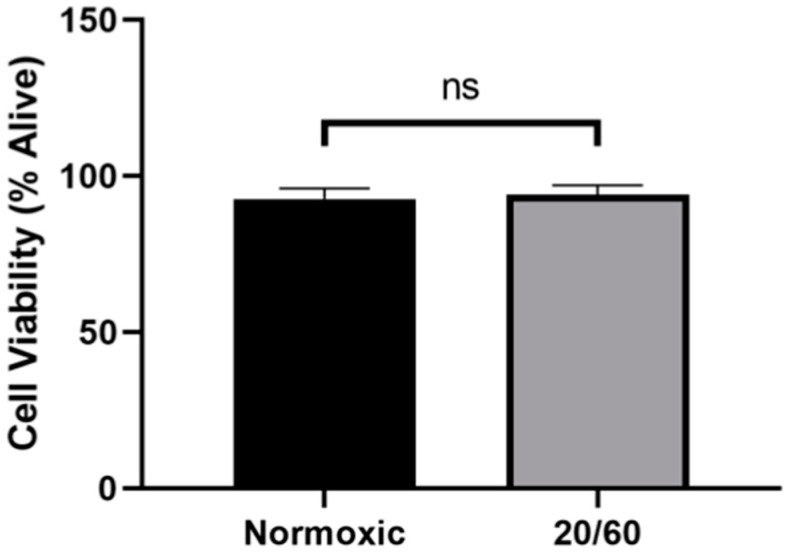
Oxygen-glucose deprivation/reperfusion (OGD/R) does not alter the viability of U251-MG cells. U251-MG cells underwent OGD for 20 min, followed by reperfusion for 60 min. There was no significant difference between the viability of normoxic control U251-MG cells and OGD/R-treated U251-MG cells (n = 3; unpaired student *t*-test). ns denotes no significance.

We first sought to determine whether our model of OGD/R resulted in increased cell death compared to normoxic controls. There was no significant difference in cell viability by trypan blue exclusion in the OGD/R-exposed cells compared to the normoxic control (Figure 1), confirming that our in vitro model of ischemia/reperfusion is not lethal and any differences in protein levels are not a result of cell death.

### 2.2. MnTMPyP Pretreatment Did Not Prevent Agonist-Induced Internalization of AMPARs

**Figure 2:**
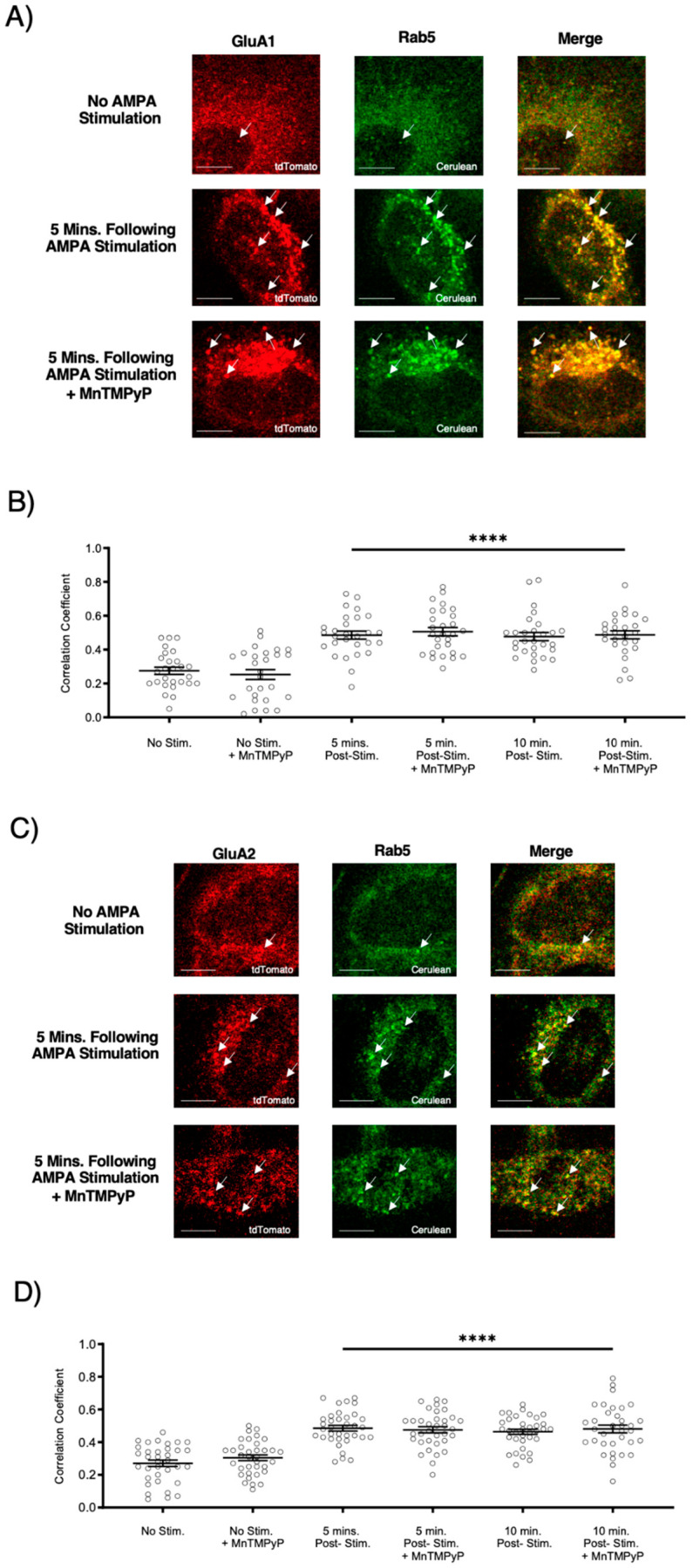
Pretreatment with Mn(III)tetrakis(1-methyl-4-pyridyl)porphyrin (MnTMPyP) did not prevent agonist-induced internalization of AMPARs. U251-MG cells transfected with either GluA1-tdTomato or GluA2-tdTomato and Rab5-cerulean, an early endosomal marker, and stimulated with AMPA agonist [100 µM]. (**A**,**B**) Increased colocalization between GluA1 and Rab5, and GluA2 and Rab5, respectively. This indicates internalization following agonist stimulation, which was unaffected by pretreatment with MnTMPyP, a superoxide scavenger. (**C**,**D**) are quantifications of (**A**,**B**), respectively (n = 30). **** *p* < 0.0001; ANOVA with Tukey’s post hoc test comparing agonist conditions to corresponding controls (no agonist stimulation ± MnTMPyP). Data are expressed as mean ± SEM. Scale bar is 100 µm. Arrows indicate colocalization.

To examine whether our superoxide dismutase mimetic, MnTMPyP, prevents agonist-induced internalization, we first exposed U251-MG cells to 3 min of agonist stimulation (100 µM AMPA; 100 µM cyclothiazide) in the presence or absence of MnTMPyP (100 µM). Following the 3 min of AMPA/cyclothiazide stimulation, cells were fixed at 5- or 10-min time-points. There was an increase in colocalization between GluA1 and Rab5, and GluA2 and Rab5 following AMPA stimulation (Figure 2). This increased colocalization indicates that GluA1 and GluA2 AMPAR subunits were internalized and presented within Rab5-positive early endosomes. Agonist-induced internalization of GluA1 and GluA2 in Rab5-positive endosomes was not antagonized by MnTMPyP.

**Figure 3:**
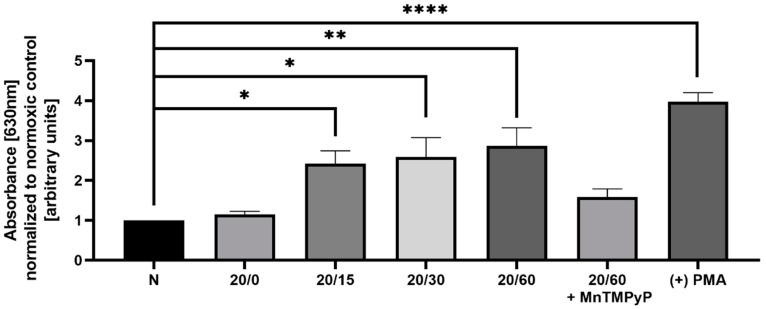
Pretreatment of OGD/R-exposed U251-MG cells with MnTMPyP antagonized reactive oxygen species (ROS) generation. Spectrophotometric quantification of ROS following either OGD/R or normoxic conditions with or without pre-treatment of MnTMPyP (100 µM) utilizing nitroblue tetrazolium chloride (NBT), to examine ROS production (n = 3). Phorbol 12-myristate 13-acetate (PMA; 1 µM for 15 min) was used as a positive control. * *p* < 0.05; ** *p* < 0.01; **** *p* < 0.0001; ANOVA with Tukey’s post hoc test comparing OGD/R conditions to normoxic control ± MnTMPyP). Data are expressed as mean ± SEM.

OGD/R has been previously shown to produce reactive oxygen species (ROS) in hippocampal and cortical neurons [22], and we wanted to examine whether we observe a similar effect in our model system. Utilizing a nitroblue tetrazolium (NBT) assay, we determined that U251-MG cells produce ROS in a time-dependent manner with OGD/R exposure. Phorbol 12-myristate 13-acetate (PMA) activates NADPH oxidase resulting in increased ROS production [38] and was utilized as a positive control for the NBT assay. ROS production following OGD/R was first observed at reperfusion time-point 15 min, and maximally produced with 60 min of reperfusion. Pretreatment with MnTMPyP ameliorated this OGD/R-induced increase in ROS (Figure 3). Based upon previous results [39,40] within our lab, we sought to determine whether superoxide production had a direct effect on AMPAR subunit endocytic trafficking following OGD/R.

### 2.3. Pretreatment with MnTMPyP Attenuated the OGD/R-Induced Internalization of GluA1 and GluA2 AMPAR Subunits

**Figure 4:**
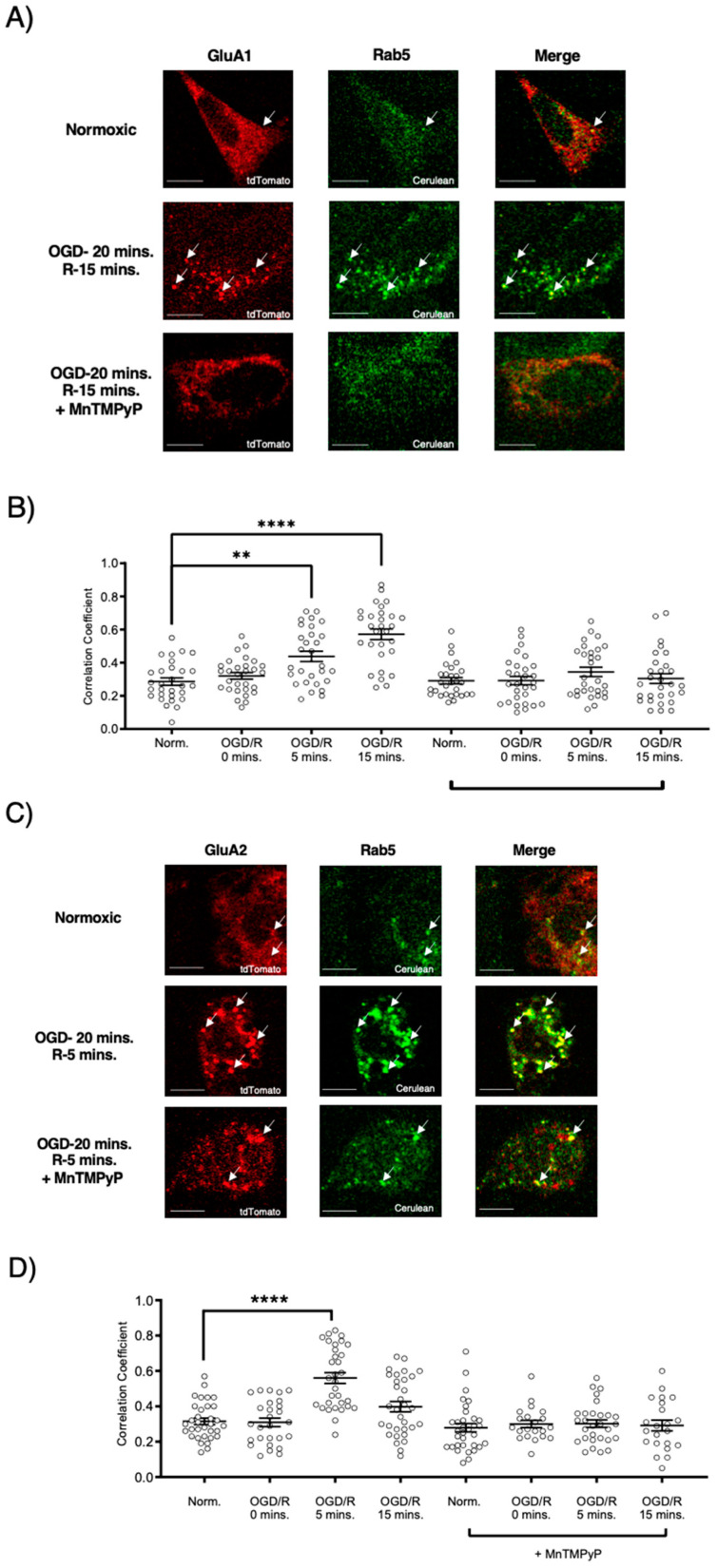
MnTMPyP prevented OGD/R-induced internalization of GluA1 and GluA2 AMPAR subunits. U-251 MG cells transfected with either GluA1-tdTomato or GluA2-tdTomato, and Rab5-cerulean, an early endosomal marker, and exposed to either normoxic or OGD/R conditions. (**A**) Increased colocalization between GluA1 and GluA2, indicating internalization, maximally at OGD/R-15 min; the effect was ameliorated with pretreatment of MnTMPyP, a superoxide scavenger. (**B**) Quantification of (**A**) microscopy (n = 30). (**C**) Increased colocalization between GluA2 and Rab5, indicating internalization, maximally at OGD/R-5 min; the effect was ameliorated with pretreatment of MnTMPyP. (**D**) Quantification of (**C**) microscopy results (n = 30). ** *p* < 0.01; **** *p* < 0.0001; ANOVA with Tukey’s post hoc test comparing OGD/R conditions to the corresponding control (normoxic ± MnTMPyP). Data are expressed as mean ± SEM. Scale bar is 100 µm. Arrows indicate colocalization.

Since our model system, U251-MG cells, produced ROS during OGD/R, we wanted to determine whether pretreatment with a superoxide scavenger, MnTMPyP, affected the endocytic trafficking of AMPAR subunits, GluA1 and GluA2. Following OGD/R, GluA1 and GluA2 subunits were both highly colocalized with Rab5-positive early endosomes. Interestingly, the time-points of GluA1 and GluA2 internalization differed with OGD/R. GluA1 was highly colocalized with Rab5 at OGD/R-5 and OGD/R-15-min time-points. GluA2 colocalized with Rab5-positive early endosomes solely at the OGD/R-5-min time-point, indicating that GluA2 moved quickly within the endocytic pathway from an early endosome to a sorting endosome, or a Rab7-positive late endosome. GluA1 and GluA2 transiently transfected in U251-MG cells internalized following OGD/R exposure. To determine whether oxidative stress played a role in this internalization, we pretreated the U251-MG cells with MnTMPyP. Pretreatment with MnTMPyP attenuated the OGD/R-induced colocalization of both GluA1 and GluA2 with Rab5-positive early endosomes (Figure 4).

### 2.4. GluA1 and GluA2 AMPAR Subunits Did Not Undergo OGD/R-Induced Degradation in the Presence of MnTMPyP

**Figure 5:**
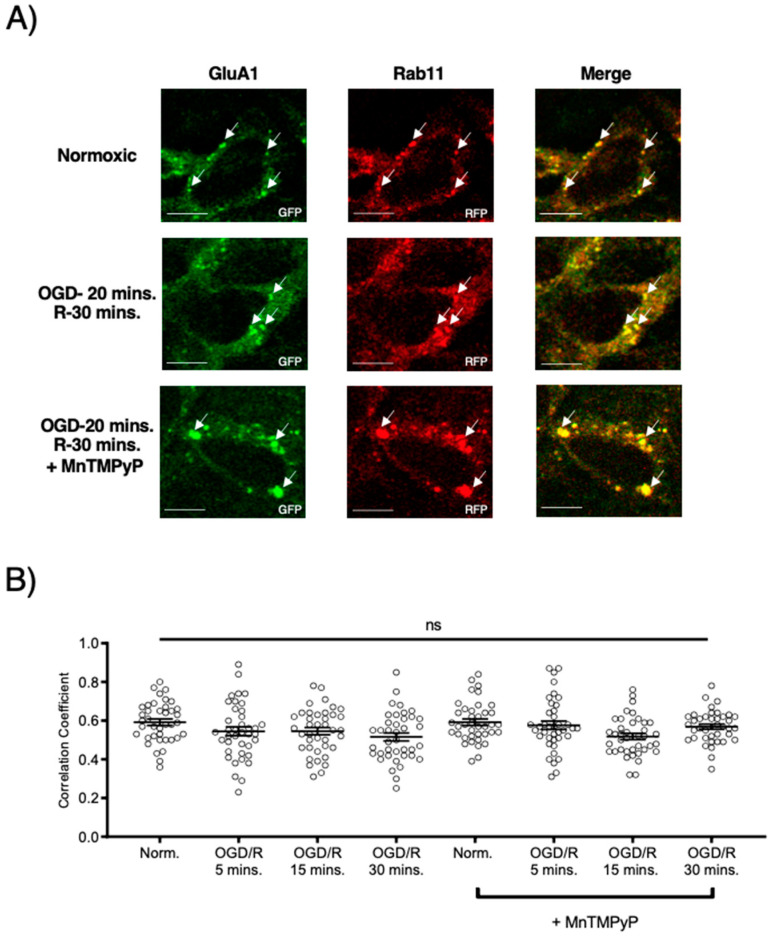

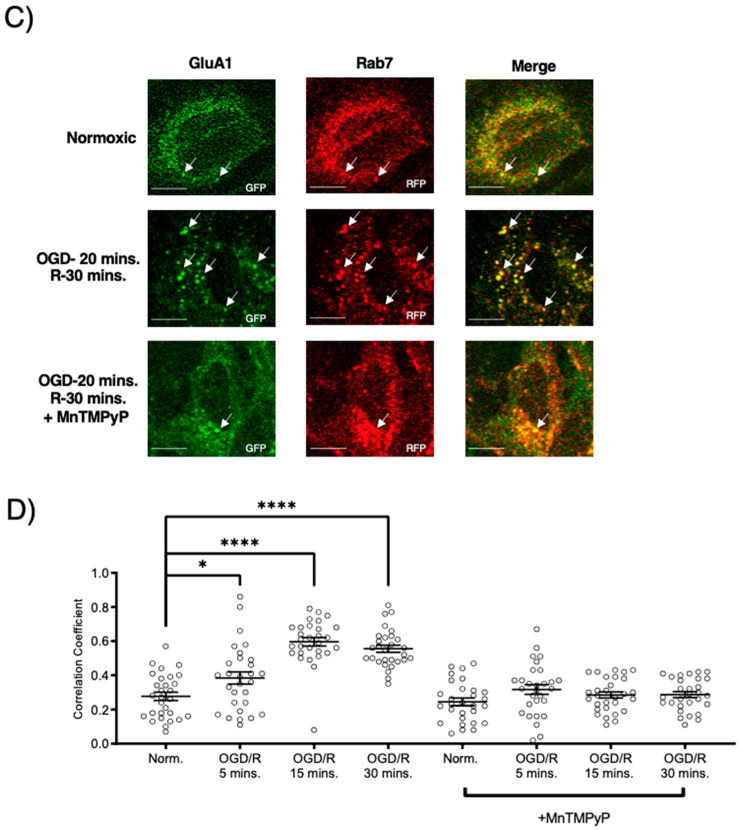
MnTMPyP prevented OGD/R-induced Rab-7, late endosomal sorting of GluA1 AMPAR subunits. GluA1 was present in Rab11-positive endosomes under all conditions. U-251 MG cells transfected with GluA1-eGFP and either Rab11-RFP, a recycling endosome marker, or Rab7-RFP, a marker for late endosomes, and exposed to either normoxic or OGD/R conditions. (**A**) High colocalization between GluA1 and Rab11 under both normoxic and OGD/R condition, unaffected by MnTMPyP treatment. (**B**) Quantification of (**A**) microscopy experiments (n = 30). (**C**) Increased colocalization between GluA1 and Rab7, indicating lysosomal degradation, maximally at OGD/R-15 min; the effect was ameliorated with pretreatment of MnTMPyP. (**D**) Quantification of microscopy (n = 30) results from (**C**). * *p* < 0.05; **** *p* < 0.0001; ns denotes no significance; ANOVA with Tukey’s post hoc test comparing OGD/R conditions corresponding to control (normoxic ± MnTMPyP). Data are expressed as mean ± SEM. Scale bar is 100 µm. Arrows indicate colocalization.

**Figure 6:**
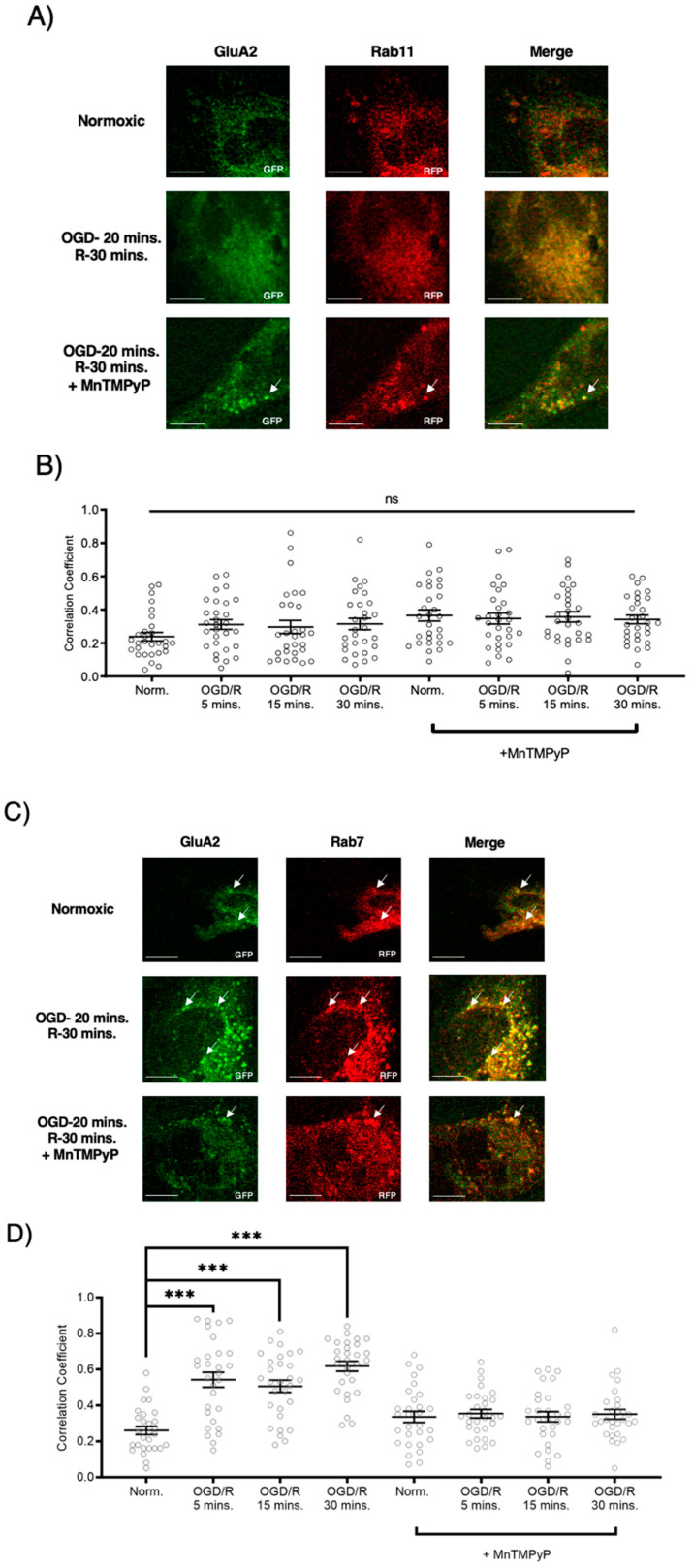
MnTMPyP prevented OGD/R-induced Rab-7, late endosomal sorting of GluA2 AMPAR subunits. GluA2 was not present in Rab11, recycling endosomes under any conditions. U-251 MG cells transfected with GluA2-eGFP, and either Rab11-RFP, a recycling endosome marker, or Rab7-RFP, a marker for late endosomes, and exposed to either normoxic or OGD/R conditions. (**A**) GluA2 did not colocalize with Rab11 under normoxic nor OGD/R conditions. (**B**) Quantification of microscopy (n = 30) results from (**A**). (**C**) Increased colocalization between GluA2 and Rab7, indicating lysosomal degradation, maximally at OGD/R-30 min; the effect was ameliorated with pretreatment of MnTMPyP. D) Quantification of microscopy (n = 30) results from (**C**). *** *p* < 0.001; ANOVA with Tukey’s post hoc test comparing OGD/R conditions to corresponding control (normoxic ± MnTMPyP). Data are expressed as mean ± SEM. Scale bar is 100 µm. Arrows indicate colocalization.

Following GluA1 and GluA2 AMPAR subunit internalization to Rab5-positive endosomes, experiments were performed to examine whether GluA1 or GluA2 AMPAR subunits are trafficked to Rab11-positive recycling endosomes in cells subjected to OGD/R. GluA1 remained highly colocalized with Rab11-positive recycling endosomes under both normoxic and OGD/R conditions. Treatment with MnTMPyP had no effect on this colocalization under normoxic nor OGD/R conditions (Figure 5A,C). Conversely, GluA2 was not highly colocalized with Rab11-positive recycling endosomes under any conditions, with or without MnTMPyP treatment. (Figure 6A,C). It is likely that there was a recycling pool of GluA1 homomeric AMPARs under both basal and pathologic conditions, but not GluA2-containing AMPARs.

To examine whether AMPAR subunits were being trafficked to the degradative pathway following OGD/R, we examined their colocalization with Rab7-positive late endosomes destined for lysosomal degradation. Both GluA1 and GluA2 subunits were highly colocalized with Rab7-positive late endosomes following OGD/R, likely indicating they were fated for degradation (Figure 5B,D and Figure 6B,D). Pretreatment with MnTMPyP, scavenging superoxide, prevented GluA1 and GluA2 AMPAR subunits being sorted to Rab7-positive late endosomes. This may occur because MnTMPyP treatment prevents the AMPAR subunits from internalizing with OGD/R or it prevents GluA1 and GluA2 subunits from trafficking to Rab7-positive endosomes with OGD/R exposure.

**Figure 7:**
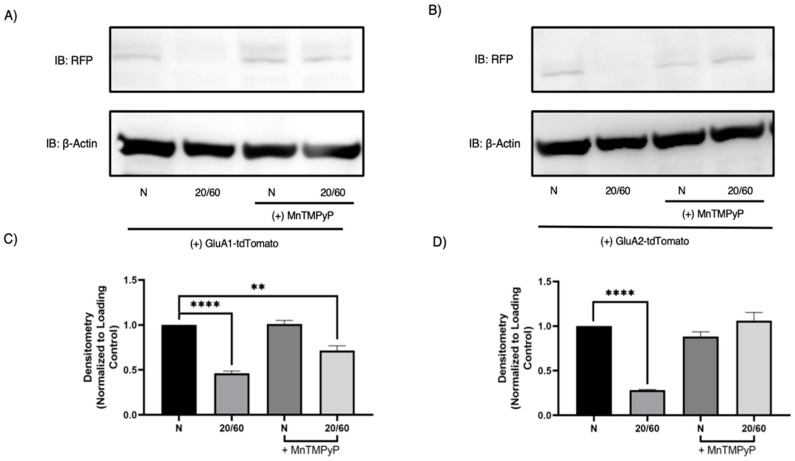
MnTMPyP prevented OGD/R-induced degradation of GluA1 and GluA2 AMPAR subunits. U-251 MG cells transfected with GluA1-tdTomato or GluA2-tdTomato and exposed to either normoxic or OGD/R conditions. (**A**) Representative Western blot demonstrating that pretreatment with MnTMPyP ameliorated OGD/R-induced decrease in total GluA1 protein levels. (**C**) Quantification (**A**) of total GluA1 protein levels normalized to β-Actin (n = 3). (**B**) Representative Western blot illustrating that pretreatment with MnTMPyP ameliorated OGD/R-induced decrease in total GluA2 protein levels. (**D**) Quantification (**B**) of total GluA2 protein levels normalized to β-Actin (N= 3). ** *p* < 0.01; **** *p* < 0.0001; ANOVA with Tukey’s post hoc test comparing OGD/R conditions to corresponding control (normoxic ± MnTMPyP). Data are expressed as mean ± SEM.

To further confirm that GluA1 and GluA2 subunits are degraded with OGD/R, we performed Western blot analysis to examine their respective protein levels. As expected, both GluA1 and GluA2 are degraded in a time-dependent manner with OGD/R. GluA1 protein levels decreased at OGD/R-30-min and maximally at OGD/R-60-min time-points. GluA2 protein levels decreased at the OGD/R-60-min time-point. Pretreatment with MnTMPyP prevented the OGD/R-induced degradation of GluA1 partially, and GluA2 completely (Figure 7).

## 3. Discussion

In this study, we have demonstrated that ischemic/reperfusion-induced internalization and subsequent endocytic trafficking of GluA1 and GluA2 AMPAR subunits to late endosomes is mediated in an oxidative stress signaling pathway. The loss of GluA2-containing AMPARs at the plasma membrane following ischemia/reperfusion occurs in vulnerable areas of the brain, such as the hippocampus, leading to delayed neuronal death [9]. The subunit composition switch from GluA2-containing, Ca^2+^-impermeable AMPARs to GluA2-lacking, Ca^2+^-permeable AMPARs occurs via the internalization and degradation of the GluA2 subunit [41], transcriptional and translational downregulation of GluA2 levels [16], and the increase in GluA2-lacking AMPARs at the plasma membrane [14]. In this study, we have examined the endocytic trafficking pathways of both GluA1 and GluA2 AMPAR subunits following OGD/R and the role of oxidative stress in mediating these processes.

It is well-studied that oxidative stress exacerbates cell death following ischemia/reperfusion [39,40,42,43,44,45]. There are three distinct temporal oxidative stress mechanisms that contribute to neuronal injury following ischemia/reperfusion. With hypoxic onset, mitochondria generate an initial burst of ROS followed closely by xanthine oxidase activation. Once reperfusion occurs, NADPH oxidase produces a large burst of ROS in a calcium-dependent manner [22]. In this study, we observed a robust increase in ROS production during the reperfusion phase of OGD/R in U251-MG cells. Astrocytes do express NADPH oxidase [46], so it is possible that the large increase in ROS is due to activation of NADPH oxidase in U251-MG cells. Alternatively, there could be delayed ROS production from mitochondria and/or xanthine oxidase. Further studies using pharmacological inhibitors and/or genetic tools are needed to identify the source of ROS in our U251-MG OGD/R model system.

This study is the first to indicate that endocytic trafficking of GluA1 and GluA2 subunits can be modulated with ROS following OGD/R. Utilizing MnTMPyP, a superoxide dismutase mimetic, we identified ROS-mediated trafficking of GluA1 and GluA2 with OGD/R that is distinct from agonist-induced internalization. MnTMPyP treatment had no effect on agonist-induced internalization of GluA1 and GluA2 subunits, but attenuated both the internalization and degradation of GluA1 and GluA2 subunits with OGD/R.

**Figure 8:**
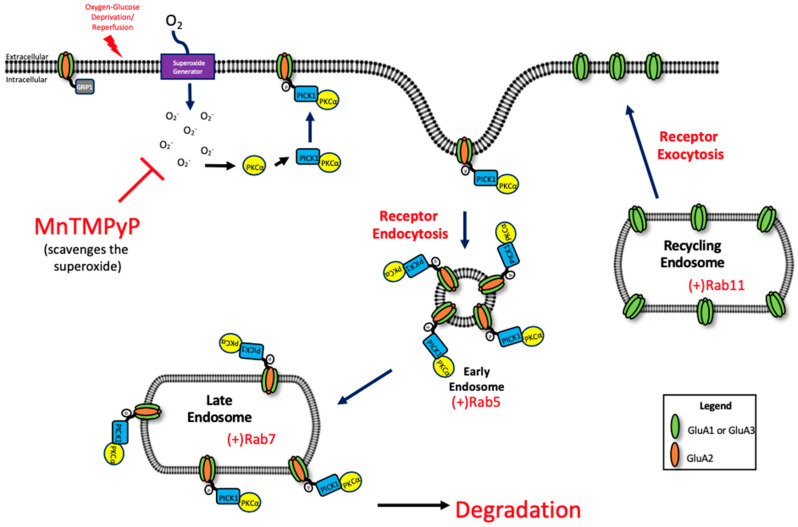
Potential mechanism of oxidative stress-mediated trafficking of GluA1 and GluA2 AMPAR subunits following OGD/R.

Under physiological conditions, endocytosis of GluA2 is mediated by protein kinase C alpha (PKCα)-dependent phosphorylation of GluA2 Ser880 residue. PKCα is activated by increased intracellular calcium and is redox-sensitive [47,48,49]. Further studies are needed to examine the relationship between ROS and PKCα activation. Upon activation, PKCα translocates the plasma membrane by protein interacting with C kinase 1 (PICK1) [50], where it phosphorylates GluA2 [51]. The phosphorylation of GluA2 at the Ser880 residue, and increased association with PICK1, increases the internalization of GluA2-containing AMPARs [19,52] thus reducing the surface population of AMPARs. This event allows for a transient increase in calcium permeable AMPARs at the plasma membrane, which is a critical component for LTP under physiologic conditions [53], but is harmful when uncontrolled during pathophysiological conditions like ischemia/reperfusion [13] (Figure 8).

The present study examined the effect of superoxide on the internalization, degradation, and recycling of GluA1 and GluA2 AMPAR subunits with OGD/R in U251-MG cells. The superoxide scavenger, MnTMPyP, prevented both the internalization of both GluA1 and GluA2 receptor subunits following OGD/R. In the absence of superoxide, the degradation of GluA1 was partially rescued with OGD/R, whereas the degradation of GluA2 was completely prevented with MnTMPyP treatment. Interestingly, GluA1 was present within Rab11-positive recycling endosomes under both normoxic and OGD/R conditions, with MnTMPyP having no effect. This suggests that there is a pool of GluA1 homomeric AMPARs that is unique to GluA1, as GluA2 was not present in Rab11-positive endosomes under any conditions studied.

The recycling of GluA1 homomeric AMPARs to the plasma membrane, a critical component of LTP [53], is a tightly regulated process. Activity-dependent endocytic sorting of GluA1 depends on phosphorylation [54] at the Ser845 residue by PKA, which promotes receptor insertion and decreases receptor endocytosis [55]. The fate of internalized GluA1-containing AMPARs to either Rab7-containing endosomes, fated for degradation, or Rab11-containing endosomes, fated for recycling, depends upon phosphorylation [56]. It has also been reported that ubiquitination of GluA1 facilitates agonist-induced endocytosis [57] and lysosomal targeting [58]. GluA1-containing, GluA2-lacking AMPARs are recruited to the plasma membrane surface during LTP by palmitoylation of A-kinase anchoring protein 150 (AKAP150), a scaffold that binds to and regulates GluA1 phosphorylation and trafficking [59]. Upon AMPA agonist stimulation, myosin Vb captures and mobilizes Rab11-positive, recycling endosomes for AMPAR insertion [60]. The mechanisms for GluA1 trafficking during LTP are well understood, but whether all these mechanisms are conserved during pathologic ischemic/reperfusion injury remains to be determined. It is particularly interesting that GluA1 homomeric AMPAR subunits are present within Rab11-positive endosomes, but GluA2 homomeric AMPAR subunits are not during OGD/R. This suggests that there is an unknown differential trafficking mechanism between GluA1 and GluA2 with regards to receptor recycling during OGD/R.

We utilized an immortalized cell line in this study, which is a study limitation. It is unclear whether these results are reproducible in different cell lines, in primary cultures of neuronal cells, or in vivo, and further studies will need to be conducted to confirm these results.

This study is the first to examine how oxidative stress mediates endocytic trafficking of GluA1 and GluA2 AMPAR subunits following OGD/R, but further studies are needed to fully characterize the role of superoxide in mediating the endocytic trafficking and degradation of the GluA1 and GluA2 AMPAR subunits with OGD/R.

## 4. Materials and Methods

### 4.1. Cell Culture

U251-MG cells, an immortalized human astrocytoma cell line, were maintained in DMEM supplemented with 10% fetal bovine serum, 1× penicillin/streptomycin, 1× MEM nonessential amino acids, and 1× sodium pyruvate. All reagents for cell culture were purchased from Gibco (Gaithersburg, MD, USA). Once cells reached 75% confluency, they were passaged.

### 4.2. Transfection

U251-MG cells were transiently transfected with GluA1-tdTomato, GluA1-eGFP, GluA2-tdTomato, or GluA2-eGFP, and either Rab5-cerulean, Rab7-RFP, or Rab11-RFP. Cells were transfected using Lipofectamine 2000 in Opti-mem media (Fisher Scientific, Pittsburgh, PA, USA) for 4 h before the transfection media was replaced with complete DMEM media. Experiments were performed 48 h following transfection.

### 4.3. AMPA Stimulation

Transfected U251-MG cells (75,000 cells/well on 6-well plate) were exposed to saturating conditions of AMPA (100 µM) [61], cyclothiazide (100 µM) [62] to prevent AMPAR desensitization, and MnTMPyP (100 µM) [63,64] as previously indicated for 3 min before being removed and replaced with complete medium. U251-MG cells were fixed with 4% paraformaldehyde at time-points 5 and 10 min. Cells were washed twice with phosphate buffered saline (PBS; Fisher Scientific, Pittsburgh, PA, USA) and mounted on slides for microscopy analysis.

### 4.4. Oxygen Glucose-Deprivation/Reperfusion (OGD/R)

Transfected U251-MG cells were exposed to deoxygenated artificial cerebrospinal fluid (aCSF) without glucose (aCSF-glucose; NaCl [124 mM], KCl [2.5 mM], NaHCO_3_ [26 mM], NaHPO_4_ [1.25 mM], CaCl_2_ [2.5 mM], MgCl_2_ [1.5 mM], sucrose [10 mM] or d-glucose [10 mM] for reperfusion conditions (aCSF+glucose)) in a hypoxic chamber for 20 min. aCSF-glucose was deoxygenated with nitrogen for 18 h prior to the experiment to ensure hypoxic conditions. aCSF-glucose was replaced with aCSF+glucose and cells were reperfused at various time-points: 0, 5, 15, 30, or 60 min. Experiments were conducted in the presence of absence of MnTMPyP [100 µM], a superoxide dismutase mimetic. U251-MG cells were either prepared for Western blotting analysis or fluorescent microscopy. Normoxic controls were time-matched to the longest OGD/R time-point.

### 4.5. Microscopy

Following OGD/R or AMPA stimulation experiments, transfected U251-MG cells were imaged via confocal microscopy (60X objective), Olympus FluoView1000/IX81 confocal microscope system (Olympus Corporations of the Americas Headquarters, Center Valley, PA, USA) and colocalization coefficients (Pearson’s Correlation Coefficient) were obtained using FIJI software (freely available from National Institutes of Health, Bethesda, MD, USA). All microscopy and data analyses were conducted in a blind manner.

### 4.6. Western Blotting

Protein concentration was determined using a bicinchoninic acid assay (BCA; Thermo Fisher Scientific, Waltham, MA, USA) and samples were heated at 100 °C in LDS/reducing agent buffer (Thermo Fisher Scientific, Waltham, MA, USA) and resolved via sodium dodecyl sulfate-polyacrylamide gel electrophoresis (SDS-PAGE). Samples were then transferred to a nitrocellulose membrane (Bio-Rad, Berkeley, CA, USA). Blots were blocked for one hour at room temperature with 5% (*w*/*v*) non-fat dry milk in tris buffered saline, 0.1% (*v*/*v*) tween-20, pH 7.5 (TBS-T). After blocking, blots were incubated with the primary antibody overnight at 4 °C at the concentration indicated. Blots were incubated with the secondary antibody for 1 h at room temperature. The following antibodies were used: anti-RFP (1:500; Invitrogen, Waltham, MA, USA), anti-β-Actin (1:10,000; Cell Signaling, Danvers, MA, USA), anti-mouse (1:2000; Cell Signaling, Danvers, MA, USA), and anti-rabbit (1:2000; Cell Signaling, Danvers, MA, USA). Immunoreactive bands were visualized and captured with a Fuji imaging system using enhanced chemiluminescence. Bands were analyzed using Fuji Image-Gauge software (Fujifilm North America Corporation, Valhalla, NY, USA).

### 4.7. Trypan Blue Exclusion

Following OGD/R, U251-MG cells were incubated in trypsin/EDTA (0.05%) and centrifuged at 1000× *g* for 10 min. The cell pellet was resuspended in complete medium and trypan blue (1:1 dilution). Cell viability was determined using trypan blue exclusion on Countess II Cell Counter (Thermo Fisher Scientific, Waltham, MA, USA).

### 4.8. NBT Assay

U251-MG cells were plated at 5 × 10^4^ in 6 well plates and, once they reached 70% confluency, were used for experiments. NBT assay was adapted from [65,66]. Briefly, after 4 h of serum starvation, 0.5 mg/mL NBT was added to cells and incubated for one hour. Excess NBT was washed away 3 times with warmed PBS. U251-MG cells underwent 20 min of OGD and various reperfusion time points: 0, 15, 30, or 60 min with or without MnTMPyP treatment. Phorbol 12-myristate 13-acetate (PMA; 1 µM; Sigma Aldrich, St. Louis, MO, USA) was used as a positive control. Following treatment, cells were fixed with absolute methanol, air dried, and formazan deposits were dissolved in 100% dimethyl sulfoxide and KOH [2M]. Absorbance was measured at 620 nm with Spectra Max Gemini M2 plate reader (Molecular Devices, Sunnyvale, CA, USA).

### 4.9. Data Analysis and Scientific Rigor

Either Student’s *t*-test or one-way ANOVA with Tukey’s post hoc test were conducted using GraphPad Prism 8 software to determine statistical significance. All imaging and analyses were performed blindly.

## Data Availability

All data are present within the manuscript or available by request to corresponding author, Darrell A. Jackson (darrell.jackson@wsu.edu).

## Abbreviations

aCSF	Artificial cerebrospinal fluid
aCSF+glucose	aCSF supplemented with glucose
aCSF-glucose	aCSF supplemented with sucrose
AMPAR	(-amino-3-hydroxy-5-methyl-4-isoxazolepropionic acid receptor
BCA	Bicinchoninic Acid Assay
DOAJ	Directory of Open Access Journals
DND	Delayed Neuronal Death
LTD	Long-Term Depression
LTP	Long-Term Potentiation
MDPI	Multidisciplinary Digital Publishing Institute
MnTMPyP	Mn(III)tetrakis(1-methyl-4-pyridyl)porphyrin
NBT	Nitroblue Tetrazolium
NMDAR	N-methyl-D-aspartate receptor
OGD/R	Oxygen Glucose-deprivation/Reperfusion
PBS	Phosphate Buffered Saline
PICK1	Protein Interacting with C Kinase 1
PKCα	Protein Kinase C alpha
PMA	Phorbol 12-myristate 13-acetate
ROS	Reactive Oxygen Species
SDS-PAGE	Sodium Dodecylsulfate-Polyacrylamide Gel Electrophoresis
TBS-T	Tris Buffered Saline + Tween
